# Spontaneous pneumothorax and pneumomediastinum in patients with COVID‐19: A case series from Iran

**DOI:** 10.1002/ccr3.5355

**Published:** 2022-02-11

**Authors:** Zeynab Yassin, Mohammad Ebrahimian, Omid Motamedi, Hale Afshar, Oldooz Aloosh, Shirin Sayyahfar, Donya Maleki, Mojtaba Ghorbi

**Affiliations:** ^1^ 440827 Antimicrobial Resistance Research Center Iran University of Medical Sciences Tehran Iran; ^2^ Medical Student Faculty of Medicine School of Medicine Hazrat‐ e Rasool General Hospital Iran University of Medical Sciences Tehran Iran; ^3^ Hazrat‐ e Rasool General Hospital Iran University of Medical Sciences Tehran Iran; ^4^ Department of Internal Medicine School of Medicine Hazrat‐ e Rasool General Hospital Iran University of Medical Sciences Tehran Iran; ^5^ 440827 Research Center of Pediatric Infectious Diseases, Institute of Immunology and Infectious Diseases Iran University of Medical Sciences Tehran Iran; ^6^ 440827 Department of Infectious and Tropical Diseases School of Medicine Iran University of Medical Sciences Tehran Iran; ^7^ Department of Anesthesiology Ahvaz Jundishapur University of Medical Sciences Ahvaz Iran

**Keywords:** coronavirus, COVID‐19, pneumomediastinum, pneumonia, spontaneous pneumothorax

## Abstract

Here, we report six cases of spontaneous pneumothorax and pneumomediastinitis in patients with COVID‐19 in Iran, which were treated with different drugs such as hydroxychloroquine, sofosbuvir, atazanavir, and remdesivir as antiviral agents. Despite the differences in the type of drugs, pneumothorax occurred in all patients.

## Graphical abstract

- Chest CT can provide useful information in patients with COVID‐19‐associated pneumonia.
- Pneumothorax and pneumomediastinum may develop in COVID‐19‐associated pneumonia due to alveolar damage.
- Pneumothorax and pneumomediastinum may be the first symptom of COVID‐19 or a late complication.
- Pneumothorax and pneumomediastinum may appear in the patients with different antiviral therapy.

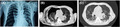

## 1 INTRODUCTION

Coronavirus disease 2019 (COVID‐19) pandemic has now infected more than 273,000,000 people and claimed more than 5,000,000 lives worldwide according to Johns Hopkins University data.^1^ The first infected patient was admitted to Wuhan Central Hospital in China on December 26, 2019, suffering severe respiratory syndrome.^1^ It is reported that the incidence of COVID‐19 is lower in pediatric patients (2.4% of all reported cases) compared to adults.^2^, ^3^ Although we now have a better understanding of the clinical manifestations of this virus and know many of its modes of transmission, we are constantly learning of potential new symptoms and long‐term effects associated with this illness.

In this article, we report a series of six cases of patients in Iran with spontaneous pneumothorax and pneumomediastinitis that tested positive for COVID‐19.

### 1.1 Case 1

**FIGURE 1:**
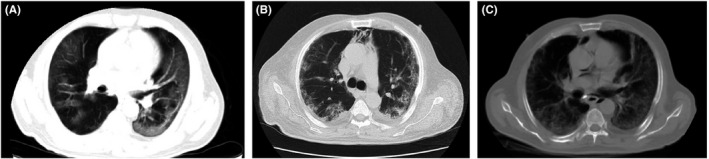
Chest CT scans of case 1

A 58‐year‐old non‐smoker man was referred to the hospital on March 10, 2020, with shortness of breath, fever, and a dry cough. Upon physical examination, his temperature was 38.5°C, his heart and respiration rates were 105 and 25 per minute, respectively, and his oxygen (O_2_) saturation was 80% on room air. His past medical history was unremarkable. We performed a real‐time reverse transcriptase‐polymerase chain reaction (RT‐PCR) test for SARS‐CoV‐2 on his oropharyngeal swab sample, which had a negative result. The chest CT scan revealed bilateral multifocal peripheral ground‐glass opacities that were more prominent in lower parts with subpleural parenchymal bands and increased vascular markings. An incidental finding of sliding hiatal hernia also was seen, which was typical for COVID‐19 patients with co‐rads 5 (very highly suspicious) (Figure 1A). Laboratory data are shown in Tables 1 and 2.

**TABLE 1:** Hematology and biochemistry laboratory data on admission

patient	WBC /mm3	PMN /mm3	Lymph /mm3	Hb g/dL	Hct %	PlT /mm3	PT second	PTT second	INR	BUN mg/dl	Cr mg/dL	Na meq/L	K meq/L	SGOT IU/L,	SGPT IU/L	LDH U/L	CRP mg/L
1	10,500	8000	1100	12.5	36.9	224000	13	36	1	14	1.2	136	4.6	28	18	391	48
2	4300	3200	688	12.4	38.1	200,000	13	30	1	26	1.5	134	4.6	84	29	844	48
3	7000	5500	750	13.2	39.1	427,000	16	34	1	17	1.4	134	4.6	64	29	440	NM
4	8000	6500	1100	13	39	185,000	13	35	1	7	0.5	131	4.2	36	49	491	NM
5	7200	5400	1440	17	51	190,000	12,	32	1.5	14	1.6	141	4.5	40	20	616	48
6	7600	6308	912	14.9	44.7	317,000	16	33	1.5	15	1.5	135	4.6	58	26	1117	12

Abbreviation: NM, not mentioned.

**TABLE 2:** Blood gas data on admission

Patient	PH	PCO2 mmHg	PO2 mmHg	HCO3 meq/L
1	7.36	47.7	28.1	27.3
2	7.37	41.5	22.4	24.4
3	7.45	41.5	19.3	29.5
4	7.41	37	30	24
5	7.40	36	37.9	22
6	7.43	39	22.4	26

Erythrocyte sedimentation rate (ESR) for the patient was 62 mm/h, and the patient was admitted due to COVID‐19‐associated pneumonia. He was treated with 400 mg of hydroxychloroquine 400 mg. He was then placed on 400/100 mg of lopinavir/ritonavir (every 12 h) for 10 days. Since there was no improvement with the course of treatment and because of sustained hypoxemia and tachypnea, the patient was subsequently placed on 400/60 mg of sofosbuvir–daclatasvir at (daily) for ten days, The patient was then transferred to ICU due to persistent hypoxemia and was supported by non‐invasive ventilation with bi‐level positive airway pressure (BIPAP). He also received 4 mg of dexamethasone three times a day for ten days and intravenous immunoglobulin (IVIG) at 30 g daily for three days. His chest CT scan was repeated two days after BIPAP administration and showed bilateral multifocal peripheral ground‐glass opacities and consolidations that were more prominent in lower parts and mild left pleural effusion with superimposed pneumomediastinum (Figure 1B). General condition and O_2_ saturation improved through seven days of BIPAP administration. Two months following admission, his pneumomediastinum was resolved (Figure 1C), and the patient was discharged with conservative care, and his O_2_ saturation on room air was 84% on the discharge day. Although the patient's PCR test for COVID‐19 was negative at admission (likely due to false‐negative results), his serology test for COVID‐19 (IgM and IgG) that was performed 16 days after admission was positive.

### 1.2 Case 2

**FIGURE 2:**
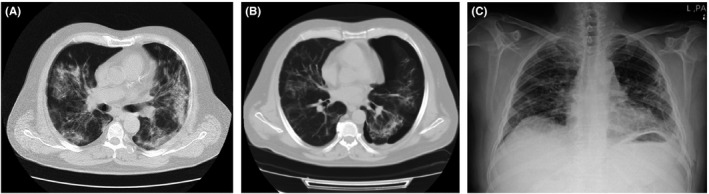
Chest CT scans and chest X‐ray of case 2

A 59‐year‐old non‐smoker man was referred to the hospital on March 20, 2020, with shortness of breath, fever, persistent dry cough, myalgia, and malaise that had persisted since a week prior to admission. Upon physical examination, his oral temperature was 38°C, his heart and respiratory rates were 110 and 28 per minute, respectively, and his O_2_ saturation was 82% on room air. The patient's past medical history was unremarkable. Due to the current ongoing pandemic, COVID‐19 was suspected, and an oropharyngeal swab was taken and sent for evaluation by RT‐PCR test. His CT scan revealed bilateral multifocal patchy ground‐glass infiltrations that were more prominent in peripheral zones of lower parts and with subpleural bands, typical for COVID‐19 patients with co‐rads 5 (highly suspicious) (Figure 2A).

However, evaluation for SARS‐CoV‐2 was negative at admission. The results of his laboratory tests are shown in Tables 1 and 2. He was admitted and treated with 200 mg of hydroxychloroquine (BID) and 400/60 mg of sofosbuvir–daclatasvir daily for ten days. After 72 hours and due to low O_2_ saturation, he was also placed on 4 mg of dexamethasone at every 8 hours for five days. Serology testing for COVID‐19 (IgM and IgG) 12 days after admission was positive. General condition of the patient improved during his hospital stay, and his O_2_ saturation was elevated to 92% on room air at the time of discharge one month after admission.

Twenty‐five days after discharge, the patient returned to the hospital with chest discomfort, dyspnea, and an O_2_ saturation of 95% on room air. Chest CT imaging (Figure 2B) showed bilateral multifocal patchy ground‐glass infiltrations that were more prominent in peripheral of lower parts. Left‐sided pneumothorax was also identified in the image. The patient underwent tube thoracostomy and was discharged after five days with full recovery from pneumothorax (Figure 2C).

### 1.3 Case 3

A 64‐year‐old non‐smoker man referred to the hospital on April 11, 2020, with shortness of breath, fever, myalgia, nonproductive cough, and dyspnea. In physical examination, his temperature was 37.8°C, and his heart and respiratory rates were 100/min and 23/min, respectively. His O_2_ saturation on room air was 87%. The patient's past medical history was unremarkable. A chest CT scan was done. Due to the current ongoing pandemic, COVID‐19 was suspected; the RT‐PCR test for SARS‐CoV‐2 was performed on a nasopharyngeal swab.

**FIGURE 3:**
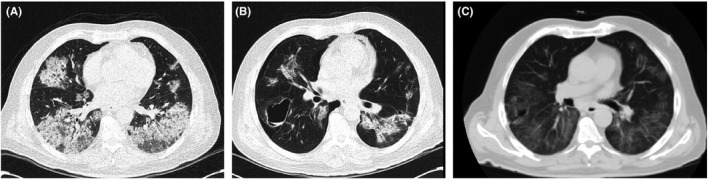
Chest CT scans of case 3

The chest CT scan revealed bilateral multifocal ground‐glass opacities and consolidations in peripheral that were more prominent in lower parts and increased vascular markings that were typical for COVID‐19 patients with co‐rads 5 (highly suspicious) (Figure 3A).

ESR for the patient was 96 mm/h, and D‐dimer was 2986 ng/ml. The patient was admitted and treated with 200 mg of hydroxychloroquine (BID) and 400/60 mg of sofosbuvir–daclatasvir daily for ten days. Following the course of therapy, the general conditions of the patient improved, and the patient was discharged after 10 days from admission with an O_2_ saturation of 92% on room air. The PCR test for SARS‐CoV‐2 performed on the nasopharyngeal sample taken on admission was negative. This may have been due to false‐negative results in PCR tests. Laboratory data for this patient are shown in Tables 1 and 2.

This patient was referred to the hospital seven days after discharge with chest pain and dyspnea and O_2_ saturation 95% on room air. His chest CT scan also revealed bilateral multifocal patchy ground‐glass infiltrations and consolidations in peripheral that were more prominent in lower parts and with air cyst 55 × 35 mm in the apical segment of right lower lobe and right‐sided pneumothorax (Figure 3B). Due to the negative PCR test result on the previous admission, we performed serology testing for COVID‐19, which revealed that both IgM and IgG were positive. After 12 h of observation after admission, a CT scan revealed that the size of the pneumothorax did not increase. According to surgical consultation, the patient did not need tube thoracostomy and was discharged without any symptoms. Follow‐up imaging (two weeks later) showed full recovery from pneumothorax (Figure 3C).

### 1.4 Case 4

A 67‐year‐old man with a history of smoking (20 packs/year) referred to the hospital on May 27, 2020, with sudden shortness of breath and chest discomfort. Upon physical examination, his temperature of the patient was 37.6°C, his heart and respiratory rates were 115 and 28 per minute, respectively, and his O_2_ saturation was 88% on room air. He had no history of contacts with COVID‐19‐suspected persons in the family, but he had mild pharyngitis and malaise a week prior to the arrival at the hospital.

**FIGURE 4:**
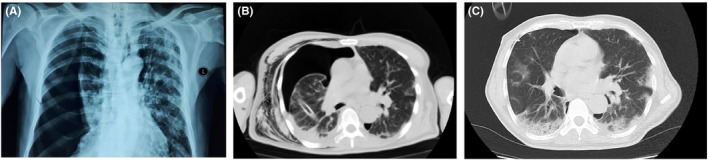
Chest CT scans and chest X‐ray of case 4

His chest X‐ray revealed right‐sided massive pneumothorax (Figure 4A). A pigtail pleural catheter was immediately inserted in the right hemithorax, and a chest CT scan was performed that showed bilateral centri and pan acinar emphysema that were more prominent in the upper parts with bolus formation in apical of right upper lobe. He also had bilateral multifocal peripheral patchy ground‐glass infiltrations and consolidations that were more prominent in lower parts with mild right‐sided pneumothorax in right side pleural space. These findings were highly suspicious for COVID‐19 infection with co‐rads 4 (Figure 4B).

PCR test for SARS‐CoV‐2 for this patient was negative, but his serology test for IgM and IgG antibodies against COVID‐19 on the seventh day after admission was positive. He was admitted and treated with 200 mg hydroxychloroquine (BID) and 300/100 mg atazanavir/ritonavir daily. The pigtail catheter was replaced by a tube thoracostomy. The general condition of the patient improved daily, and on the 11th day following admission, the patient's pneumothorax was resolved (Figure 4C), and the patient was discharged. The patient's laboratory data are shown in Tables 1 and 2.

### 1.5 Case 5

**FIGURE 5:**
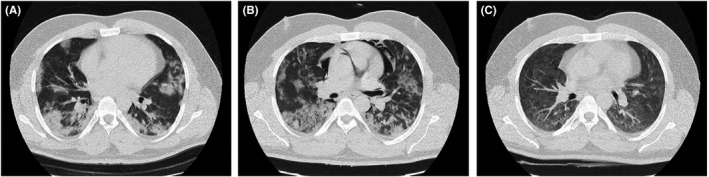
Chest CT scans of case 5

On July 27, 2020, a 34‐year‐old non‐smoker male patient with no underlying disease was presented to our emergency department complaining of fever, cough, myalgia, and shortness of breath. His vital signs were within normal limits (blood pressure: 110/70 mmHg, heart rate: 82/min, respiratory rate: 16/min, and temperature: 37.2°C). He had an O_2_ saturation of 88% without an O_2_ mask on room air. His chest CT scan on admission showed peripheral multifocal ground‐glass opacities, which affected multiple lobes of both lungs (Figure 5A). He had a lymphopenia (lymph count: 1444), a CRP of 48 mg/L, and an ESR of 25 mm/h. His SARS‐CoV‐2 PCR test performed on nasopharyngeal specimens was positive. Data are shown in Tables 1 and 2.

The patient was placed on 300/100 mg of atazanavir/ritonavir daily and 200 mg of hydroxychloroquine (BID), followed by 44 mcg of interferon every other day and 4 mg of dexamethasone every 8 hours due to hypoxia. On day 5 following admission, and despite his treatment, his O_2_ saturation level decreased to 81%. A repeated chest CT scan revealed pneumomediastinum (Figure 5B). However, he continued to experience hypoxia (O_2_ saturation of 80% on day 8th following admission). The patient subsequently received three liters of plasma exchange, and his clinical status gradually improved. The patient was discharged two weeks after admission with an acceptable general condition. Upon follow‐up investigations, a chest CT image three weeks after discharge showed no signs of pneumomediastinum (Figure 5C).

### 1.6 Case 6

A 53‐year‐old non‐smoker man with no significant medical history was evaluated for dry cough and shortness of breath three days before admission. Furthermore, he experienced fever and chills, weakness, myalgia, nausea, vomiting, and anorexia 11 days before evaluation. On admission, he had a blood pressure of 110/75 mmHg, a heart rate of 80/min, tachypnea (respiratory rate: 25/min), and a fever (temperature: 38°C). His O_2_ saturation on room air was 75%. His chest CT scan revealed bilateral multifocal ground‐glass opacities, which were more prominent in peripheral zones. The results of his laboratory tests showed a lymphopenia (lymph count: 912), CRP: 12 mg/L, IL‐6: 294, and LDH: 1117 U/L. He tested positive for SARS‐CoV‐2 by PCR. Laboratory data are presented in Tables 1 and 2.

**FIGURE 6:**
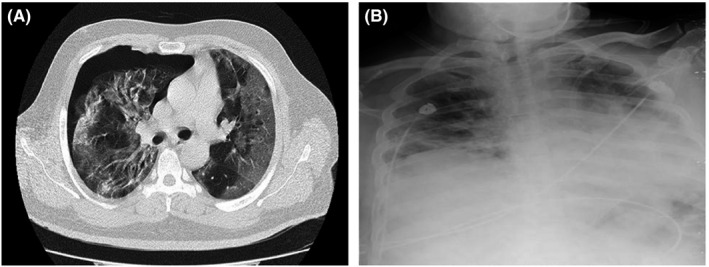
Chest CT scan and chest X‐ray of case 6

On admission, the patient received 300/100 mg of atazanavir/ritonavir daily 44 mcg of interferon every other day, and 4 mg of dexamethasone every 8 h. He was admitted to ICU and was administered 100 mg of remdesivir daily. On day 3 following admission, his O_2_ saturation decreased to 67%. We repeated a chest CT scan on day 5 following admission. The scan revealed a moderate right‐sided pneumothorax (Figure 6A), and we placed a chest tube in the right hemithorax. On day 10 following admission, the patient developed bradycardia and tachypnea, and his O2 saturation level decreased to 80% on a reservoir O_2_ mask. He experienced severe respiratory distress. His chest X‐ray showed patchy bilateral opacification with reticular opacities that were more prominent in lower parts with bilateral blunt costophrenic angles (Figure 6B). He was immediately intubated and, despite aggressive treatment measures, became worse and died a few hours later.

## 2 DISCUSSION

According to the World Health Organization (WHO), the main symptoms of COVID‐19 are fever, dry cough, shortness of breath, fatigue, muscle pain, arthralgia, headache, and sore throat.^4^, ^5^ The China Centers for Disease Control and Prevention (China CDC) identified 44,672 COVID‐19 positive patients until February 14, 2020, from which 965 cases (2.2%) were under 20, and the mortality rate was 0.1%. 77.8% of the patients were between 30 and 69 years old. Significant signs and symptoms include fever (87.9%), dry cough (67.7%), fatigue (38.1%), sputum production (33.4%), shortness of breath (18.6%), and sore throat (13.9%).^5^ Similarly, our patients’ symptoms were mostly fever, cough, shortness of breath, and chest pain. Our patients had no comorbidities, previous operation, and history of pneumothorax.

A chest CT scan and a RT‐PCR test for SARS‐CoV‐2 were recommended by the Chinese National Health Commission for diagnosis of COVID‐19. The latter was used for definitive diagnosis of COVID‐19 infection, but in the present study, all RT‐PCR results were negative because of false‐negative results in PCR tests, although all cases had positive serology (IgM & IgG) for SARS‐CoV‐2. In the Tao Ai et al. study, the sensitivity of RT‐PCR and chest CT scan was 97% and 88%, respectively.^6^

Present findings highlight the importance of a chest CT scan for the screening of patients suspected of COVID‐19. In COVID‐19‐associated pneumonia, the typical early finding in a chest CT scan is bilateral multilobar ground‐glass opacification with a peripheral or posterior distribution. This finding occurs in the early stage of COVID‐19‐associated pneumonia due to alveolar septal inflammation.^7^ Present findings suggest that imaging methods serve as a standard diagnostic approach in symptomatic cases with negative RT‐PCR results. Known typical features of COVID‐19 on initial chest CT scan are bilateral multilobar ground‐glass opacification with a peripheral or posterior distribution, apparent in the outer lateral zone of lungs. Secondary spontaneous pneumothorax is one of the poor prognostic factors, which occurs in interstitial lung disease such as idiopathic pulmonary fibrosis (IPF).^8^ More reticular involvement in HRCT predicts increase possibility of pneumothorax occurrence in this population.^8^

About one percent of patients with COVID‐19 have pneumothorax.^9^ In our patients, the ground‐glass opacity was accompanied by pneumothorax in three patients and by pneumomediastinum in one patient. There were no underlying lung disease, smoking, or structural abnormality in the body that would predispose our patients to pneumothorax formation, except in one case. In a study done by Wang et al., spontaneous pneumothorax, pneumomediastinum, and subcutaneous emphysema presented simultaneously in a 62‐year‐old Chinese man with bilateral multiple ground‐glass opacities in his chest CT scan who had a positive test for SARS‐CoV‐2.^9^ Multiple factors may contribute to the spontaneous pneumothorax and pneumomediastinum, including tobacco smoking, age, gender, low body mass index, height, prolonged cough, strenuous exercise, and comorbidity such as chronic obstructive pulmonary disease (COPD). The patient's data are summarized in Table 3. None of the patients were vaccinated against COVID‐19 at the time of this research. Vaccination probably can change clinical presentation and complication of COVID‐19, and it should be checked. Spontaneous pneumomediastinum, usually a rare condition, refers to alveolar rupture due to an increase in intrathoracic pressure followed by air dissection through the bronchovascular sheath into the mediastinum. It has been reported that pneumothorax may develop in COVID‐19‐associated pneumonia due to alveolar damage.^10^, ^11^ Spontaneous rupture of a subpleural bulla is another cause of primary spontaneous pneumothorax.^9^

**TABLE 3:** Summary of the patient's data

Patient	Age	Gender	Smoking status	Mechanical ventilation	How many days passed from admission when complication occurred	Therapeutic agent
1	58	Male	Non‐smoker	No	22	H^(I)^ + K^(II)^ + D^(III)^
2	59	Male	Non‐smoker	No	25	H + S^(IV)^ + D
3	64	Male	Non‐smoker	No	17	H + S
4	67	Male	smoker	No	0 (on admission)	H + A^(V)^
5	34	Male	Non‐smoker	No	5	H + A + D
6	53	Male	Non‐smoker	Yes	5	A + R^(VI)^ + D

Note

(I): hydroxychloroquine

(II): Kaletra (Lopinavir/ritonavir)

(III): Dexamethasone

(IV): Sofosbuvir/daclatasvir

(V): Atazanavir/ritonavir

(VI): Remdesivir

Spontaneous pneumothorax might also be related to deep airway and alveolar damage caused by COVID‐19, particularly the distal alveoli with mucus‐like exudation. Narrowing and distortion of the bronchioles secondary to inflammation and secondarily, the valve mechanism could cause pulmonary bullae. Intrapulmonary pressure increase due to coughing may also lead to bullae rupture and secondary pneumothorax.^12^ Although a rare feature, pneumothorax has also been reported not only in patients with COVID‐19 but also in those with SARS and MERS diseases.^13^

One study reported that strong cough attacks could cause widespread alveolar damage secondary to a sudden increase in alveolar pressure and pneumothorax.^10^ In another study on 919 COVID‐19 positive patients, ground‐glass densities were reported in 88% of cases, and 87.5% of parenchymal attitudes were observed to be bilateral, while none of them encountered pneumothorax.^14^ In severe acute respiratory failure syndrome, sudden alveolar pressure increase may cause interstitial emphysema and air leak, leading to the development of mediastinal emphysema.^15^ In our hospital, the following regiment protocols were prescribed for COVID‐19 positive patients: Kaletra + hydroxychloroquine; atazanavir + hydroxychloroquine; sofosbuvir + hydroxychloroquine; favipiravir + hydroxychloroquine; remdesivir. Three out of six patients in this study were treated with sofosbuvir + hydroxychloroquine with or without dexamethasone, two patients were treated with atazanavir + hydroxychloroquine, and one patient was treated with remdesivir. Despite the differences in the type of drugs, pneumothorax and pneumomediastinum occurred in all patients. These observations suggest that the difference in the type of these drugs may not prevent pneumothorax and pneumomediastinum.

It is likely that spontaneous pneumothorax and pneumomediastinum could be a complication of lung involvement in COVID‐19, at presentation, during admission, and in convalescence duration. Clinicians should be mindful of these symptoms and consider this differential diagnosis such as CT scan when a patient has respiratory symptoms during this COVID‐19 pandemic.

## CONFLICTS OF INTEREST

All authors declare that they have no conflict of interest.

## AUTHOR CONTRIBUTIONS

All authors were involved in the writing, revision, and final review of the manuscript.

## ETHICAL APPROVAL

For publishing this case report, we asked Rasoul‐Akram hospital ethical committee for approval. We informed the patient about the process of publishing a case report, and he signed the consent form.

## CONSENT

Written informed consent was obtained from the patient to publish this report in accordance with the journal's patient consent policy.

## Data Availability

The data that support the findings of this study are available on request from the corresponding author. The data are not publicly available due to the fact that their containing information could compromise the privacy of research participants.
